# RQ18 Peptide Exerts Anti-Melanoma Effects via Apoptosis Induction

**DOI:** 10.3390/ph19101521

**Published:** 2026-09-25

**Authors:** Claudiane Vilharroel Almeida, Ana Cristina Jacobowski, Caio Fernando Ramalho de Oliveira, Luís Henrique de Oliveira Almeida, Camila de Oliveira Gutierrez, Tamaeh Monteiro-Alfredo, Lucas Rodrigues de Lima, Thais de Andrade Farias Rodrigues, Rodrigo Juliano Oliveira, Octávio Luiz Franco, Marlon Henrique Cardoso, Ana Paula de Araújo Boleti, Maria Lígia Rodrigues Macedo

**Affiliations:** 1 Programa de Pós-Graduação em Bioquímica e Biologia Molecular, Instituto de Biociências (INBIO), Universidade Federal de Mato Grosso do Sul, Campo Grande 79070-900, MS, Brazil; 2 Laboratório de Purificação de Proteínas e suas Funções Biológicas (LPPFB), Faculdade de Ciências Farmacêuticas, Alimentos e Nutrição (FACFAN), Universidade Federal de Mato Grosso do Sul, Campo Grande 79070-900, MS, Brazil; 3 S-Inova Biotech, Programa de Pós-Graduação em Biotecnologia, Universidade Católica Dom Bosco, Campo Grande 79117-900, MS, Brazil; 4 Centro de Estudos em Células Tronco, Terapia Celular e Genética Toxicológica (CeTroGen), Faculdade de Medicina (FAMED), Universidade Federal de Mato Grosso do Sul, Campo Grande 79070-900, MS, Brazil; 5 Centro de Análises Proteômicas e Bioquímicas, Programa de Pós-Graduação em Ciências Genômicas e Biotecnologia, Universidade Católica de Brasília, Brasília 71966-700, DF, Brazil; 6 Programa de Pós-Graduação em Ciências Ambientais e Sustentabilidade Agropecuária, Universidade Católica Dom Bosco, Campo Grande 79117-900, MS, Brazil

**Keywords:** anticancer peptide, melanoma, selectivity cytotoxicity, apoptosis, molecular dynamics

## Abstract

**Background/Objectives:** The rise in cancer cases is a major global public health problem. Treating the disease and maintaining health generate high economic and social costs. In recent years, treatment resistance has become a challenge, reinforcing the need for new anticancer agents. In this context, anticancer peptides (ACPs) stand out for their selective interaction with cancer cell membranes. This study aimed to evaluate the activity of the RQ18 peptide against cancer cells. **Methods:** The cytotoxicity of RQ18 was determined by the MTT assay in murine melanoma (B16F10-Nex2), human melanoma (Sk-mel-28 and A375), breast cancer (MCF-7), cervical cancer (HeLa), murine macrophage (RAW 264.7), and human fibroblast (FN1). Caspase-3/7 activity was further evaluated in B16F10-Nex2, Sk-mel-28, and A375 melanoma cells to investigate the involvement of apoptotic pathways. In addition, intracellular oxidative stress was assessed using the DCFH-DA probe in these three melanoma cell lines. In B16F10-Nex2 cells, cellular morphological changes were examined by microscopy, while apoptosis-associated events were characterized using the markers MitoTracker Deep Red, and NucBlue, complemented by flow cytometry analyses. In addition, molecular dynamics simulations were employed to investigate its interactions with lipid membranes. **Results:** The peptide exhibited anticancer activity, with IC_50_ values of 4.4, 11.58 and 59.99 µM·mL^−1^, against cells B16F10-Nex2, Sk-mel-28 and A375, respectively. Compared with non-cancerous cells, RQ18 preferentially affected cancer cells, especially B16F10-Nex2. In contrast, the peptide was nontoxic to healthy cells, thus indicating selectivity for cancer cells. Morphological analysis revealed alterations compatible with apoptosis, including cell rounding and chromatin condensation. RQ18 increased caspase-3/7 activity in B16F10-Nex2, Sk-mel-28, and A375 cells and induced intracellular oxidative stress, as indicated by increased DCFH-DA fluorescence. Flow cytometry data confirmed the induction of apoptotic cell death in B16F10-Nex2 cells. Caspase activation, detected by a caspase-3/7 assay in three melanoma cell lines, together with flow cytometry data, supported the involvement of apoptotic cell death. Molecular dynamics simulations suggest that the peptide interacts stably with cancer cell-like membranes and retains an α-helical conformation during membrane association. **Conclusions:** In summary, the RQ18 peptide demonstrates preferential anticancer activity, particularly against melanoma cells, supporting further investigation of its therapeutic potential.

## 1. Introduction

Cancer remains one of the world’s most significant public health challenges [1,2,3]. This group of diseases is characterized by the excessive proliferation of cells in the body, which cannot be effectively regulated [4]. These cells multiply uncontrollably without following the normal processes of cell growth and death. Moreover, they invade parts of surrounding organs and the body through a process called metastasis, a key factor in cancer progression and the onset of severe complications [5,6].

Despite significant advances in conventional therapeutic strategies, including chemotherapy, radiotherapy, immunotherapy and surgery, many types of cancer still have high recurrence and mortality rates [7,8,9,10]. Among such therapies, chemotherapy remains a primary choice due to its non-invasive and anti-metastatic nature [11]. However, the limited effectiveness of some treatments is often associated with the intrinsic or acquired resistance of cancer cells, as well as severe side effects that compromise patients’ quality of life [12,13]. These limitations are particularly pronounced in aggressive or advanced cancers, where the response to treatment is often unsatisfactory [14,15].

The emergence of resistance to anticancer drugs is a complex process that arises from changes in drug targets in cancer cells [16]. The mechanisms of resistance to multiple drugs and the lack of cancer-specific targeting result in side effects for patients [17]. Resistance to conventional chemotherapy is a significant cause of treatment failure [12]. Despite this, chemotherapy remains the most established treatment for eliminating rapidly dividing cancer cells [18]. However, most drugs used to treat cancer have low cell selectivity, leading to indiscriminate killing of both cancer cells and normal cells [19]. Therefore, current research focuses on identifying compounds that selectively target tumor cells, while sparing normal tissues [10,18].

Anticancer peptides (ACPs) have emerged as promising candidates in the pharmaceutical industry, offering advantages over conventional agents [8,19]. These peptides can be identified from various natural sources or developed through rational design strategies, often assisted by computational tools [20]. They are classified as a subclass of antimicrobial peptides (AMPs), which also exhibit antitumor activity [21,22]. Several AMPs exhibit selective cytotoxicity against cancer cells, a property attributed to differences in the composition and physicochemical properties of tumor cell membranes compared to healthy cells [23,24,25,26,27]. The surface of cancer cells is characterized by a net negative charge, resulting from the exposure of anionic molecules, such as phosphatidylserine, in addition to alterations in lipid composition and increased membrane fluidity [21,28,29,30]. These characteristics favor electrostatic interactions with cationic peptides [21,25,28]. The AMP sequences described to date exhibit high variability, but together they share recurring structural properties, such as net positive charge and amphipathic helical organization at the membrane interface, sometimes modulated by flexible regions. The intensity and mode of interaction of these peptides with biological membranes depend on a combination of factors, including chain length, secondary structure, charge, hydrophobicity, and amphipathic character [31,32,33,34].

ACPs frequently exhibit rapid action against tumor cells, with less impact on healthy cells [20,21]. Their mechanisms of action involve multiple pathways, including membrane destabilization and permeabilization, induction of apoptosis, inhibition of angiogenesis, and modulation of the immune response [33]. Among these processes, apoptosis and necrosis stand out, frequently associated with loss of cell membrane integrity [34]. The biological activity of these peptides is closely related to their primary sequence and structural organization, which confer amphipathic, hydrophobic, and cationic properties [34,35,36]. In particular, hydrophobicity plays a central role in the interaction with the lipid bilayer, allowing greater attraction to the hydrophobic core of the membrane and favoring the formation of pores or channels [37]. This attraction is associated with cytotoxic efficacy and the interaction profile with tumor membranes [8]. Despite advances in the development of ACP, limitations remain regarding a detailed understanding of their mechanisms of action, as well as obtaining molecules that combine high selectivity and low cytotoxicity in non-tumor cells. In this context, rational design-based approaches have been explored to optimize the structural and functional properties of these peptides.

In this context, the RQ18 peptide [38,39], known for its antimicrobial properties, was evaluated herein for its anticancer potential. RQ18 was initially developed through in silico approaches, guided by physicochemical properties in association with antimicrobial activity and cellular selectivity. The peptide sequence comprises 18 amino acid residues that are capable of adopting an α-helical conformation in membrane-mimicking environments. In the present study, we investigated the antitumor activity of RQ18 against different cancer cell lines, as well as its cytotoxicity, selectivity, and possible mechanisms of action. Our data revealed a high selectivity of RQ18 against cancer cells, reinforcing the potential of anticancer peptides as innovative tools in cancer treatment and supporting the advancement of targeted peptide-based therapies.

## 2. Results

### 2.1. Antimicrobial Potential of the RQ18 Peptide

The RQ18 peptide (NH_2_-RALRKALKAWRKLAKKLQ-NH_2_) sequence, described by Almeida et al. (2021) [38], was analyzed in silico to evaluate its potential anticancer properties (Table 1). Based on bioinformatic predictions, using the tools EnnaACT [40], dbAMP 3.0 [41], ACPred [42], MLACP 2.0 [10], ACPP [43] and AntiCP 2.0 [44], RQ18 exhibited a high potential for activity against cancer cells.

### 2.2. Evaluation of the Cytotoxic Potential of RQ18

The cytotoxicity of the RQ18 peptide was evaluated in cancer cell lines (B16F10-Nex2, Sk-mel-28, HeLa, MCF-7 and A375), and non-cancerous healthy mammalian cell lines (FN1 and RAW 264.7), using the MTT assay. The assay revealed a dose-dependent reduction in cell viability upon peptide treatment. The IC_50_ values were 4.4 µM·mL^−1^ for B16F10-Nex2, 11.58 µM·mL^−1^ for Sk-mel-28, 15.58 µM·mL^−1^ for HeLa, 120.6 µM·mL^−1^ for MCF-7 and 59.99 µM·mL^−1^ for A375. For the non-cancerous cell lines, the IC_50_ values were 23.08 µM·mL^−1^ for FN1 and 69.2 µM·mL^−1^ for RAW 264.7 [39], as shown in Table 2. The selectivity index (SI) was calculated as the ratio of the IC_50_ value for non-cancerous cells (RAW 264.7) to the IC_50_ value for each cancer cell line and indicated selective cytotoxicity of RQ18. SI values greater than 1 reflect preferential cytotoxicity towards cancer cells. The highest selectivity was observed for B16F10-Nex2 cells (SI = 15.73), followed by Sk-mel-28 (SI = 5.97), HeLa (SI = 4.44) and A375 (SI = 1.15). These results indicate preferential cytotoxicity of RQ18 toward several cancer cell lines, with the strongest selectivity observed for B16F10-Nex2 and Sk-mel-28 compared with RAW 264.7 cells.

Additionally, FBS contains proteins and other components that may affect the activity and availability of bioactive peptides. To evaluate this, the impact of varying FBS concentrations on the cytotoxic activity of RQ18 against B16F10-Nex2 cells was investigated. The IC_50_ values were determined in culture media supplemented with increasing concentrations of FBS. A progressive increase in IC_50_ values was observed with higher FBS content, indicating reduced apparent peptide efficacy. The IC_50_ increased from 4.4 µM·mL^−1^ in serum-free medium to 6.1 µM·mL^−1^ in medium supplemented with 2.5% FBS, 10.16 µM·mL^−1^ with 5% FBS, and 18.73 µM·mL^−1^ with 10% FBS. These findings indicate that serum components reduce the apparent cytotoxic activity of RQ18 in a concentration-dependent manner. This effect may reflect reduced peptide availability through peptide–protein interactions, proteolytic degradation, or a combination of these mechanisms.

### 2.3. RQ18 Increases Caspase-3/7 Activity in Melanoma Cells

Caspase-3/7 activity was evaluated in B16F10-Nex2, Sk-mel-28, and A375 melanoma cells following RQ18 treatment using the CaspaTag™ FLICA assay. The unstained control was used to determine basal fluorescence in the absence of the FLICA reagent, whereas the FLICA-stained control consisted of untreated cells incubated with the reagent. As shown in Figure 1, RQ18 treatment increased fluorescence associated with caspase-3/7 activity in B16F10-Nex2 and Sk-mel-28 cells, whereas an increase in lower intensity was observed in A375 cells. In B16F10-Nex2 cells (Figure 1A), mean fluorescence intensity increased from 0.460 RFU in the FLICA-stained control to 0.724 RFU following RQ18 treatment, corresponding to an approximately 57% increase, which suggests apoptotic signaling. In Sk-mel-28 cells (Figure 1B), RQ18 treatment increased mean fluorescence intensity from 0.432 RFU in the FLICA-stained control to 1.048 RFU, corresponding to an approximately 2.4-fold increase. In contrast, in A375 cells (Figure 1C), fluorescence increased from 0.391 RFU in the FLICA-stained control to 0.435 RFU following RQ18 treatment, representing an approximately 11% increase. These findings indicate that RQ18 is associated with increased caspase-3/7 activity in the evaluated melanoma cells, with the greatest response observed in Sk-mel-28 and B16F10-Nex2 cells.

### 2.4. RQ18 Induces Intracellular Oxidative Activity in Melanoma Cells

To investigate whether RQ18 treatment was associated with alterations in intracellular oxidative activity, B16F10-Nex2, Sk-mel-28, and A375 cells were evaluated using the fluorescent probe DCFH-DA. As shown in Figure 2, untreated cells from all three melanoma cell lines showed no detectable green fluorescence, whereas RQ18-treated cells exhibited a marked increase in DCF fluorescence. This increase was observed in all three cell lines after 1 h of treatment, consistent with an early alteration in intracellular oxidative activity following RQ18 treatment. Representative images obtained at 1 h were selected for presentation because this time point allowed clear visualization of the fluorescence response while maintaining adequate cellular density for microscopic analysis.

The increase in DCF fluorescence observed following RQ18 treatment is consistent with a perturbation of intracellular redox homeostasis in melanoma cells. Maintenance of redox balance is essential for cellular homeostasis, as excessive oxidative activity can affect critical cellular components, including lipids, proteins, nucleic acids, and mitochondria, potentially compromising cellular function and viability. Thus, the early increase in DCF fluorescence observed in B16F10-Nex2, Sk-mel-28, and A375 cells suggests that disruption of intracellular redox homeostasis may be associated with the early cellular response to RQ18 treatment.

### 2.5. Effect of RQ18 on Cancer Cell Morphology

Based on the more pronounced RQ18-induced caspase-3/7 activation observed in B16F10 cells, subsequent mechanistic experiments were performed exclusively in this cell line. Morphological alterations induced by the RQ18 peptide at its IC_50_ concentration (4.4 µM·mL^−1^) were evaluated in murine melanoma B16F10-Nex2 cells using automated live-cell imaging with the ZenCELL microscope. Multipoint images were captured every 5 min (Figure 3), revealing a gradual disruption in cellular architecture. At 0 min, cells exhibited a typical fusiform morphology, with uniform adhesion to the substrate. Following RQ18 treatment, morphological changes became apparent at approximately 60 min post-treatment. The cells progressively became rounded, reflecting a loss of adhesion, which is commonly associated with decreased viability and compromised plasma membrane integrity. Additionally, increased chromatin condensation was observed, suggesting the induction of apoptosis. More intense nuclear staining, especially after 60 min of treatment, confirmed this effect. Control cells showed no significant changes following incubation with the peptide, confirming that the RQ18 peptide induces specific morphological and nuclear alterations in melanoma cells.

### 2.6. Effect of RQ18 on Mitochondrial Potential and Nuclear Integrity

The mitochondrial viability of B16F10-Nex2 cells was assessed after treatment with the peptide at IC_50_ (4.4 µM·mL^−1^). Figure 4 shows images of murine melanoma cells from the control group and the group treated with RQ18, obtained by fluorescence microscopy after staining with NucBlue™ and MitoTracker™ Deep Red. MitoTracker™ is a membrane-permeable dye whose accumulation in mitochondria is dependent on mitochondrial membrane potential; thus, functionally intact mitochondria exhibit intense red fluorescence. NucBlue^TM^ is a DNA-specific fluorescent dye that binds preferentially to the cell nucleus, allowing visualization of nuclear morphology and identification of structural changes associated with cell death processes.

Untreated cells showed preserved nuclear morphology, with homogeneous chromatin distribution, as well as strong MitoTracker™ fluorescence, indicating maintenance of mitochondrial membrane potential and cell viability. In contrast, cells treated with RQ18 exhibited a marked reduction in red fluorescence, evidencing loss of mitochondrial membrane potential and impaired mitochondrial function, a result associated with activation of the apoptotic pathway. Additionally, the nuclei of treated cells showed increased NucBlue™ fluorescence intensity, reflecting nuclear morphological changes, such as swelling and changes in chromatin organization, compared to the control group. The reduction in mitochondrial potential, together with caspase activation observed in complementary assays, indicates that the mechanism of action of RQ18 involves the induction of apoptosis.

### 2.7. Flow Cytometry Analysis

B16F10-Nex2 cells treated with the RQ18 peptide at IC_50_ (4.4 µM·mL^−1^) were analyzed by flow cytometry using Annexin V-FITC and propidium iodide (PI) staining to distinguish between viable cells, early apoptosis, late apoptosis, and necrosis (Figure 5). Annexin V-FITC staining detects phosphatidylserine externalization, a hallmark of early apoptosis, whereas PI penetrates only cells with compromised membranes, labeling cells in late apoptosis or necrosis. The results showed that approximately 55% of the treated cells (Figure 5B) were located in the Q2 quadrant (Annexin V+/PI+), corresponding to the late apoptosis stage. These findings demonstrate that RQ18 predominantly induces apoptotic cell death in B16F10-Nex2 cells.

### 2.8. Interaction of RQ18 with Cancer Cell Membranes, as Visualized by MD Simulations

The structural behavior of RQ18 and its effects on the cancer membrane were analyzed through interactions with the POPC/POPS/POPE lipid bilayer (3:1:1) (Figure 6A). The data obtained show that the peptide interacts with and tends to penetrate the membrane surface. The interaction with the membrane showed greater stability and ensured α-helical conformational conservation throughout the simulation time. Distances ranging from approximately 0.1 to 0.3 nm were observed (Figure 6B), with the most stable conformation, indicated by the root mean square deviation (RMSD), reached at around 50 ns. Root mean square fluctuation (RMSF) analyses were also performed (Figure 6C). A similar pattern was observed in the radius of gyration (Rg) analysis, which showed no significant variation among the simulated systems (Figure 6D). These results provide initial insight into RQ18’s binding preference for cancer cell-like membranes. They qualitatively reflect observations from biological tests, supporting the hypothesis that the mechanism of action involves interaction with membranes.

## 3. Discussion

The limited number of effective cancer treatments is just one of the several factors that have reinforced the impact of this disease on public health. Chemotherapy remains one of the primary therapeutic approaches in cancer treatment; however, the side effects associated with this modality pose significant challenges to its clinical efficacy. Current treatments lack selectivity for cancer cells, leading to adverse effects that compromise the quality of life of patients and may result in treatment discontinuation. As such, the development of new cancer treatments has been directed toward the design and synthesis of selective, cytotoxic chemotherapeutic agents based on well-defined mechanisms of action [45]. ACPs are considered a new generation of promising anticancer drugs due to their mode of action, although their development requires a balance between efficacy and low toxicity [46].

In our previous study, the antimicrobial peptide RQ18 was characterized as an antimicrobial agent against pathogenic bacteria [38] and fungi [39], which motivated further investigation into its other biological activities. In the present study, the RQ18 peptide was evaluated for its potential as an ACP. Computational tools allowed us to predict the anticancer functionality of RQ18 through amino acid sequence analysis. Bioinformatics analysis previously documented RQ18 as a cationic peptide (+8 charge). It contains approximately 50% hydrophobic amino acids, is 18 residues long, is amphipathic, and adopts an α-helical conformation [38,39]. At the time, we characterized RQ18 as a potent antimicrobial peptide that acts via membrane-associated mechanisms. In the present study, we demonstrate that these same mechanisms also enable RQ18 to target cancer cells.

The anticancer effects of the RQ18 peptide were demonstrated by its potent cytotoxic activity against B16F10-Nex2, Sk-mel-28, HeLa, A375, and MCF-7 cell lines. RQ18 exhibited a remarkably low IC_50_ of 4.4 µM·mL^−1^ against murine melanoma B16F10-Nex2 cells, demonstrating dose-dependent anticancer activity. In addition to evaluating RQ18’s cytotoxic effect on cancer cells, we investigated the effect of FBS concentration in the culture medium on peptide efficacy. Results showed a gradual increase in IC_50_ values for B16F10-Nex2 cells with increasing FBS concentration, indicating that serum components may affect peptide interactions with target cells. However, even at high FBS concentrations, RQ18 remained active against B16F10-Nex2 cells, suggesting mechanisms such as membrane disruption or internalization. For comparison, the LVTX-9-C peptide, a derivative of LVTX-9, had an IC_50_ of 15.9 µM·mL^−1^ in serum-supplemented medium and 13.4 µM·mL^−1^ in serum-free medium against B16F10 cells, a subline that is less resistant than those used in our assays [47]. These results indicate that FBS can act as a barrier to peptide activity against cancer cells. Given its diverse properties, RQ18 may interact electrostatically with FBS components. Nevertheless, peptide selectivity toward cancer cells has been attributed to their preferential interaction with the negatively charged membranes of cancer cells [8].

To further investigate the cellular events underlying RQ18-induced cytotoxicity, we evaluated intracellular oxidative stress in melanoma cells. A rapid increase in DCF fluorescence was observed in B16F10-Nex2, Sk-mel-28, and A375 cells after only 1 h of RQ18 treatment, whereas untreated cells showed no detectable green fluorescence. The detection of oxidative activity across all three melanoma cell lines suggests that redox perturbation may represent an early and common event associated with RQ18 activity. Notably, increased fluorescence was observed in cells exhibiting morphological alterations, supporting a temporal association between oxidative stress and the early cellular response to RQ18. At later time points, particularly at 6 and 24 h, the marked reduction in cell number per microscopic field limited microscopic assessment. Therefore, the 1-h time point was particularly informative for characterizing the early oxidative response associated with RQ18-induced cytotoxicity in melanoma cells. Together, these findings suggest that oxidative stress may contribute to the early cellular events underlying the antitumor activity of RQ18 against melanoma cells.

The relationship between redox alterations and the anticancer activity of peptides is supported by experimental studies. The antimicrobial peptide moricin, for example, induced an increase in DCFH-DA-associated fluorescence in breast cancer cells, as assessed by fluorescence microscopy, and this effect was accompanied by mitochondrial alterations and activation of apoptotic mechanisms [48]. Particularly relevant to the present study, the antimicrobial peptide Cm-p5 induced an increase in DCFH-DA-associated fluorescence in A375 melanoma cells, with fluorescence signals observed in association with cellular morphological alterations; the authors also evaluated caspase-3/7 activity following treatment [49]. The observation of a similar response following RQ18 treatment, not only in A375 but also in B16F10-Nex2 and Sk-mel-28 cells, extends this evidence and reinforces the relevance of redox perturbation as a component of the response of melanoma cells to bioactive peptides.

In the context of RQ18, this finding becomes relevant when integrated with the other results of the present study. The oxidative alteration detected as early as 1 h was accompanied by changes in mitochondrial membrane potential and, subsequently, by increased caspase-3/7 activity, particularly in Sk-mel-28 and B16F10-Nex2 cells. This temporal sequence, particularly evident in B16F10-Nex2 cells, is consistent with a close interplay between redox imbalance and mitochondrial dysfunction, suggesting that oxidative perturbation may be associated with mitochondrial alterations that contribute to the cellular events underlying RQ18-induced cytotoxicity. However, due to the limited specificity of DCFH-DA, the increase in fluorescence should be interpreted as evidence of altered intracellular oxidative activity or redox homeostasis. This interpretation is consistent with the recommendations of Nature Metabolism, which emphasize that DCFH-DA should primarily be used as an initial approach to assess alterations in the cellular redox state, with complementary approaches required to attribute the signal to specific oxidant species [50].

The activation of the executioner caspases-3 and caspase-7 represents an event associated with the execution phase of apoptosis, although activation of these enzymes may also occur in other contexts of cellular stress responses [51,52]. In the present study, RQ18 treatment increased caspase-3/7 activity in all three melanoma cell lines evaluated, indicating the involvement of the apoptotic machinery in both murine and human melanoma models.

In B16F10-Nex2 cells, an approximately 57% increase was observed relative to the FLICA-stained control, suggesting that RQ18 promotes apoptotic signaling in this highly aggressive and metastatic murine melanoma model. Sk-mel-28 cells exhibited the most pronounced response, with approximately 2.4-fold higher activity than that observed in the control, whereas A375 cells showed a more modest increase (~11%). This difference may reflect intrinsic characteristics of each cellular model, including differences in the regulation of survival and apoptotic pathways, and emphasizes the importance of evaluating potential therapeutic compounds across multiple experimental models. In A375 cells, for example, activation of the MAPK and PI3K/AKT pathways has been associated with resistance to certain therapeutic stimuli [53]. Genetic alterations affecting pathways such as MAPK and PI3K/AKT, as well as p53 status and Bcl-2 family members, may contribute to differences in apoptotic sensitivity among melanoma models [54]. In addition, differences in apoptotic signaling, mitochondrial susceptibility, or the expression of pro- and anti-apoptotic proteins may contribute to the differential response observed among the cell lines.

These findings demonstrate that RQ18 treatment is associated with caspase-3/7 activation in murine and human melanoma models, with a particularly pronounced response in Sk-mel-28 and B16F10-Nex2 cells. Considering that B16F10-Nex2 cells exhibit characteristics associated with high proliferative capacity and aggressiveness, the increased caspase activity observed following treatment suggests that RQ18 is capable of triggering the apoptotic machinery in this cellular model.

The response observed for RQ18 is consistent with mechanisms described for other anticancer peptides. Mastoparan, a cationic and amphipathic peptide, induced death of B16F10-Nex2 cells through an apoptotic pathway associated with caspase activation, loss of mitochondrial membrane potential, and increased oxidative activity [55]. Of particular relevance, a recent study with catestatin evaluated the same three cell lines used in the present study—B16F10, A375, and Sk-mel-28—and demonstrated reduced cell viability accompanied by increased caspase-3/7 activity and induction of apoptosis [56]. The observation of increased caspase-3/7 activity across the same three cell lines in the present study further supports the relevance of this parameter for characterizing the antitumor activity of peptides against melanoma and demonstrates that the response to RQ18 is consistent with apoptotic mechanisms described for other bioactive peptides.

In the context of RQ18, the greater caspase-3/7 activation observed in Sk-mel-28 and B16F10-Nex2 cells further characterizes its anticancer activity. This finding becomes particularly relevant when integrated with the early alteration in intracellular oxidative activity, changes in mitochondrial membrane potential, and the Annexin V/PI results obtained in the present study. Collectively, these convergent findings support the involvement of apoptotic mechanisms in the cytotoxic activity of RQ18 and reinforce its potential as an anticancer peptide candidate against melanoma.

Among the melanoma cell lines evaluated, B16F10-Nex2 was selected for subsequent mechanistic investigations based on the convergence of cytotoxic, oxidative, mitochondrial, and apoptotic responses induced by RQ18. B16F10-Nex2 cells exhibited a pronounced cytotoxic response, with a low IC_50_ value of 4.4 µM·mL^−1^, together with an early increase in intracellular oxidative activity, alterations in mitochondrial membrane potential, and a 57% increase in caspase-3/7 activity following treatment. Although Sk-mel-28 exhibited a more pronounced increase in caspase-3/7 activity, the consistent occurrence of multiple RQ18-induced cellular alterations in B16F10-Nex2 provided a suitable basis for further mechanistic characterization. Moreover, the aggressive and highly proliferative phenotype of B16F10-Nex2 cells makes this model particularly relevant for investigating the antitumor effects of RQ18 in melanoma.

The cell morphology of treated cells was evaluated as an additional parameter of cytotoxicity. Anticancer compounds can induce various morphological changes in target cells that reflect their specific mechanisms of action. Early morphological changes were observed within minutes of treatment, indicating a significant impact of the peptide on cell survival, potentially linked to the initiation of apoptosis. RQ18 promoted morphological alterations over time, with the maximal effect observed at 60 min. ACPs exert cytotoxic effects on cancer cells through multiple mechanisms, including direct penetration, endocytic pathways, or pore formation [20]. Their anticancer activity is primarily determined by physicochemical properties, especially their cationic and amphipathic nature. Similarly, previously described cationic peptides, such as PEPAD [57], KI17 [58] and KW18 [59], also exhibit selectivity towards tumor cells, a property associated with their net positive charge, which favors electrostatic interactions with anionic components enriched in the cell membrane. Cancer cell membranes typically contain phosphatidylcholine (PC), sphingomyelin (SM), phosphatidylserine (PS), and phosphatidylethanolamine (PE) [60]. However, although the latter is also found in the inner membranes of healthy cells, in cancer membranes it is located in the outer layer [25]. Peptides bind selectively to negatively charged PS molecules exposed on cancer cell surfaces, leading to membrane destabilization and damage [61]. The anionic properties of these phospholipids facilitate electrostatic interactions with ACPs due to their cationic and amphipathic characteristics [62], where these features confer selectivity toward negatively charged cancer cell membranes, destabilizing them. Such electrostatic selectivity is observed in peptides such as defensins A and B [63], MPI-1 [64], and pardaxin [65], which are electrostatically attracted and form pores in cancer cell membranes. In addition to membranolytic mechanisms, many ACPs have been known to activate mitochondrial pathways, inducing apoptosis and necrosis [25,55,66,67].

In B16F10-Nex2 cells, RQ18 was observed to induce cell death by apoptosis, likely by damaging cells’ mitochondrial membranes. Numerous studies have shown that ACPs exert their cytotoxicity by damaging the mitochondrial membrane, which eventually induces apoptosis, and this is considered to be one of their primary mechanisms of action [25,68,69]. Furthermore, in other studies, some ACPs were reported to activate caspases and initiate apoptotic processes, leading to cell death; these peptides include melittin [70], SALF [71] and CNSCWSKD [72]. Caspases generally regulate apoptosis and are involved in both intrinsic mitochondrial and extrinsic death receptor pathways [73]. Moreover, the peptide Brevivin1RL1 was shown to induce caspase-dependent apoptosis and necrosis, highlighting its potential as a promising anticancer drug candidate [74].

RQ18 affected mitochondrial membrane integrity, suggesting that it acts as a trigger for apoptosis. Similar findings have been reported for peptides IK-13, LK-13, CI-15, and GI-15 [8]. However, some peptides may also initiate necrosis, which was not observed for RQ18. To elucidate the interaction of RQ18 with the cancer membrane, MD simulations were carried out using bilayers composed of POPC/POPS/POPE. The results of this simulation corroborated the experimental data, indicating a stable electrostatic interaction between the peptide and the membrane. In an MD simulation study on a POPS membrane, Herrera-León et al. [75] found that HB43 peptide mutants were able to interact with and penetrate the cell membrane [75]. This effect was caused by the electrostatic attraction of two positively charged mutants, which exerted pressure, resulting in a reduction in the area of the outer leaflet of the bilayer. MD is a powerful technique that takes into account flexibility during binding, as well as all the intermolecular interactions of the surrounding environment, i.e., water molecules, ions and lipid membranes [76]. The simulations showed that RQ18 always maintained its α-helical conformation by binding to the membrane throughout the simulation period. This binding is due to a strong electrostatic interaction and supports the hypothesis that this peptide, like other AMPs, induces cell death via apoptosis [77].

Flow cytometry analysis was used to precisely determine the type of cell death induced by treatment with RQ18. Data showed that RQ18 induced significant cell death in B16F10-Nex2 murine melanoma cells, predominantly inducing apoptosis. This apoptotic profile suggests a potential mechanism of selective and effective anticancer action, and findings corroborate previous findings of low toxicity in normal cells, reinforcing the selectivity of RQ18 for melanoma cells.

Chemotherapy is the most common cancer treatment, yet it damages both healthy and cancerous cells by inhibiting DNA replication [62]. For various reasons, chemotherapy drugs cause resistance, which results in a low success rate and an increased risk of recurrence [78]. ACPs are described as promising future chemotherapeutics due to low resistance, minimal side effects, high specificity, and appropriate solubility [17,79,80].

For therapeutic applications, antimicrobial and anticancer peptides must demonstrate selective toxicity against microbial and cancer cells, while sparing normal cells [81,82]. The cationic and amphipathic properties of peptides likely contribute to selectivity, as the more negatively charged surfaces of pathogenic membranes promote peptide interaction more than neutral mammalian membranes [83]. Selectivity in cancer cells is also attributed to the abundant negatively charged phosphatidylserine, facilitating preferential peptide accumulation on cancer cell surfaces [68].

In vitro analyses carried out with normal cells, such as murine macrophages (RAW 264.7) and human fibroblasts (FN1), indicated low cytotoxicity of the peptide, with IC_50_ values of 69.2 and 23.08 µM·mL^−1^, respectively. The safety of RQ18 is a consequence of its selective profile, probably due to its preferential affinity for the anionic membranes of cancer cells, compared to normal cells. Furthermore, these evaluations are essential to demonstrate the safety of RQ18 as an innovative therapeutic alternative to the limitations of conventional chemotherapy drugs.

The anticancer activity of RQ18 against melanoma cells may be related to its structural and physicochemical characteristics, which influence its interaction with cellular membranes and downstream cellular responses. Its cationic and amphipathic properties may favor preferential association with negatively charged melanoma cell membranes, potentially contributing to membrane perturbation and triggering subsequent oxidative, mitochondrial, and apoptotic events. However, to gain further insights into the mechanism of action of RQ18, it is essential to analyze treatment-induced changes in cell morphology. This can be achieved using high-resolution imaging techniques, such as electron microscopy, in future studies. This approach will allow detailed characterization of the structural changes induced by the peptide in melanoma cells, providing further insights into its mechanism of action. Additionally, in vivo studies using appropriate melanoma models, particularly B16F10-Nex2-based models, are warranted to validate the antitumor effects observed in vitro and to investigate the pharmacokinetics, biodistribution, and safety profile of RQ18. These investigations will provide a more comprehensive understanding of RQ18 activity and its translational potential, further supporting its development as a lead candidate for peptide-based anticancer therapy.

## 4. Material and Methods

### 4.1. Peptide Synthesis

The peptide was synthesized by GenOne Biotechnology (Rio de Janeiro, Brazil), with 95% purity, using the solid-phase synthesis method. The resulting peptide was C-terminally amidated to enhance stability and bioactivity. The molecular weight of the peptide was determined by mass spectrometry analysis.

### 4.2. Bioinformatics Analysis

The RQ18 peptide sequence was analyzed using freely available online servers to identify potential anticancer properties. The following tools were employed for peptide prediction and classification for anticancer activity: Ennaact (https://research.timmons.eu/ennaact, accessed on 2 June 2026) [40], dbAMP 3.0 [41], ACPred 1.10.2 [42], MLACP 2.0 [10], ACPP (https://github.com/brsaran/ACPP, accessed on 2 June 2026) [43], and AntiCP 2.0 [44].

### 4.3. Cell Culture

The murine melanoma (B16F10-Nex2), human melanoma (Sk-mel-28 and A375), human breast cancer (MCF-7), cervical cancer (HeLa), murine macrophage (RAW 264.7) and human fibroblast (FN1) cell lines were obtained from the Cell Bank of Rio de Janeiro (BCRJ, Rio de Janeiro, Brazil). All cell lines were cryopreserved in liquid nitrogen at approximately −196 °C at the Laboratory of Protein Purification and Their Biological Functions, Federal University of Mato Grosso do Sul (UFMS, Campo Grande, MS, Brazil). The cells were cultured in Roswell Park Memorial Institute Medium (RPMI-1640, Sigma, Saint Louis, MO, USA) supplemented with 10% fetal bovine serum (FBS), 100·U mL^−1^ penicillin and 100 µg·mL^−1^ streptomycin (Gibco, Waltham, MA, USA) at 37 °C in an incubator with 5% CO_2_.

### 4.4. Cell Viability Assay

The effect of the RQ18 peptide on cell viability was assessed based on the enzymatic reduction of 3-(4,5-dimethylthiazol-2-yl)-2,5-diphenyltetrazolium bromide (MTT). Culture flasks were maintained in a 5% CO_2_ incubator at 37 °C. Cell lines B16F10-Nex2, Sk-mel-28, MCF-7, HeLa, A375, RAW 264.7, and FN1 were seeded onto 96-well plates at densities of 5 × 10^4^, 1 × 10^4^, 1 × 10^5^, 1 × 10^4^, 1 × 10^4^, 3 × 10^4^, and 5 × 10^5^ cells·well^−1^, respectively. Once cells reached 80% confluence, they were treated with different concentrations of the RQ18 peptide (1–128 μM·mL^−1^), diluted in sterile water, and incubated for 24 h in their respective culture media at 37 °C and 5% CO_2_. Controls (untreated cells) were included. After incubation, the supernatant was removed, and 100 μL of MTT solution (1 mg·mL^−1^) was added to each well. Plates were incubated for 4 h at 37 °C. Subsequently, MTT was removed, and 100 μL of dimethyl sulfoxide (DMSO) was added to solubilize the formazan crystals. Absorbance was measured at 630 nm using a Varioskan Lux microplate reader (Thermo Scientific, Vantaa, Finland), and cell viability was calculated using SkanIt 6.0 software (Thermo Scientific, Vantaa, Finland). Results were expressed as mean ± standard deviation (SD). The half-maximal inhibitory concentration (IC_50_) values were determined using GraphPad Prism 8.0 software.

### 4.5. Analysis of Cellular Morphological Changes

To evaluate morphological changes, B16F10-Nex2 cells were seeded (5 × 10^4^ cells·well^−1^) onto 24-well plates. When 90% confluence was reached, cells were treated with the RQ18 peptide at the IC_50_ concentration (µM·mL^−1^). The plate was incubated in a CO_2_ incubator at 37 °C and monitored for morphological changes over a 12-h period. The peptide’s effects were observed by monitoring cell morphology and integrity through images captured every 5 min using the 24-channel ZenCELL Owl microscope (innoME GmbH, Espelkamp, Germany).

### 4.6. Analysis of Mitochondrial and Nuclear Alterations

Cell damage caused by the RQ18 peptide involving mitochondrial and nuclear morphological changes in B16F10-Nex2 cells was assessed as described by Wodlej et al. [84]. B16F10-Nex2 cells were seeded at a density of 5 × 10^4^ cells·well^−1^ in 24-well plates containing circular coverslips, and cultured until reaching >80%. Cells were treated with 500 µL of RPMI-1640 containing RQ18 at its IC_50_ concentration and incubated for 24 h. After incubation, cells were washed with PBS and fixed with 1% (*w*/*v*) paraformaldehyde at room temperature for 10 min. For mitochondrial staining, 500 µL of RPMI-1640 and 20 µL of MitoTracker Deep Red (Molecular Probes Inc., Eugene, OR, USA) (excitation: 650 nm; emission: 668 nm) at 50 µM·mL^−1^ were added to each well and incubated for 15 min at 37 °C. For nuclear staining, 5 μL of NucBlue Live Ready Probes reagent (Molecular Probes Inc., Eugene, OR, USA) (excitation: 359 nm; emission: 461 nm) was added per well and incubated for 5 min at 37 °C. Coverslips were removed and observed under a Leica DM 2000 LED fluorescence microscope (Leica Microsystems, Wetzlar, Germany) equipped with a Leica DFC 7000 T camera (Leica Microsystems, Wetzlar, Germany). Fluorescence images were captured and analyzed using LAS V4.12 software.

### 4.7. Caspase-3/7 Activity Assay

Caspase-3/7 activity was evaluated using the CaspaTag™ Caspase-3/7 In Situ Assay Kit, Fluorescein (Sigma-Aldrich, Darmstadt, Germany) according to the manufacturer’s instructions. B16F10-Nex2, A375, and Sk-mel-28 melanoma cells were seeded into 24-well plates at a density of 1 × 10^5^ cells per well and allowed to adhere overnight under standard culture conditions (37 °C, 5% CO_2_). Following treatment with RQ18, the culture medium was replaced with fresh medium containing the CaspaTag FLICA reagent at the working concentration recommended by the manufacturer. Cells were incubated for 60 min at 37 °C, protected from light, to allow binding of the fluorescent inhibitor to activated caspase-3/7. Untreated cells without FLICA staining were used as the unstained control, whereas untreated cells with the FLICA reagent were used as the FLICA-stained control. RQ18-treated cells were also incubated with the FLICA reagent under the same conditions. After incubation, cells were washed according to the manufacturer’s protocol to remove excess unbound reagent. The fluorescence signal was quantified using a microplate spectrofluorometer at an excitation wavelength of 490 nm and an emission wavelength of 520 nm, and the results were expressed as relative fluorescence units (RFU). Increased fluorescence intensity was interpreted as increased caspase-3/7 activity and, consequently, enhanced apoptotic activity. Data were obtained from independent experiments and expressed as mean ± standard deviation (SD).

### 4.8. Evaluation of Intracellular Oxidative Activity

The intracellular oxidative activity induced by RQ18 was evaluated using the fluorescent probe 2′,7′-dichlorodihydrofluorescein diacetate (DCFH-DA). B16F10-Nex2, Sk-mel-28, and A375 cells were seeded at a density of 5 × 10^4^ cells per well in 24-well plates containing circular coverslips and cultured until reaching >80% confluence under standard culture conditions (37 °C, 5% CO_2_). The plates were maintained protected from light throughout the incubation period. Cells were then treated with RQ18 at their respective IC_50_ concentrations for 1, 2, 4, 6, and 24 h. Following each treatment period, cells were incubated with DCFH-DA at a final concentration of 20 µM for 30 min. The DCFH-DA solution was prepared in Hank’s balanced salt solution immediately before use and protected from light. A volume of 300 µL of the prepared solution was added to each well. Untreated cells were used as negative controls. After DCFH-DA incubation, no washing step was performed because of the pronounced cellular disruption observed following RQ18 treatment, which could result in additional cell loss during washing. Coverslips were removed and observed under a Leica DM 2000 LED fluorescence microscope equipped with a Leica DFC 7000 T camera. Fluorescence Images were captured and analyzed using LAS V4.12 software. Representative images were acquired for qualitative assessment of DCF fluorescence in untreated and RQ18-treated cells.

### 4.9. Cell Death Profile Analysis

The cell death profile was determined using methodology described by Pesarini et al. [85], with modifications. B16F10-Nex2 cells were seeded in 6-well plates (1 × 10^6^ cells·well^−1^) and cultured in RPMI-1640 medium supplemented with 10% FBS for 24 h. After reaching ≥ 80% confluence, cells were treated with RQ18 at the IC_50_ concentration for 24 h. Cells were washed with PBS, detached, and resuspended in binding buffer (0.1 M HEPES/NaOH (pH 7.4), 1.4 M NaCl, 25 mM CaCl_2_). The suspension was stained with fluorescein isothiocyanate (FITC)-conjugated annexin V and propidium iodide (BD Pharmingen™), according to the manufacturer’s instructions. Cells were incubated for 15 min at room temperature, and 50.000 events per sample were acquired and analyzed by flow cytometry.

### 4.10. Molecular Dynamics Simulations with Cancer Membranes

Molecular dynamics (MD) simulations were also performed to evaluate the interaction of the RQ18 peptide with a cancer cell membrane model. Peptide–membrane interaction studies used the following lipids: 1-palmitoyl-2-oleoyl-sn-glycero-3-phosphocholine (POPC), 1-palmitoyl-2-oleoyl-sn-glycero-3-phospho-L-serine (POPS), and 1-palmitoyl-2-oleoyl-sn-glycero-3-phosphoethanolamine (POPE). Simulations were conducted with a POPC/POPS/POPE bilayer (3:1:1) with an area of 75 Å^2^, using the GROMACS v.5.0.4 software package [86]. Systems were built using the CHARMM-GUI server [87] and the CHARMM36 force field. RQ18 was placed approximately 0.35 nm from the membrane. The simulations were performed at an ionic strength of 0.15 M NaCl. Water geometry was maintained using the SETTLE algorithm [88], and the LINCS algorithm was used to preserve bond lengths. Electrostatic corrections were applied using the Particle Mesh Ewald (PME) method [89] with a 1 nm cutoff radius to reduce computational time. Peptide–bilayer systems underwent ten rounds (50.000 steps) of equilibration at 310 K and 1 bar using the velocity rescaling thermostat (NVT) [90] and Parrinello-Rahman barostat (NPT) [91], respectively, for 100 ps. Systems with minimized energy and equilibrated temperature and pressure were subjected to 500 ns MD simulations using a leap-frog algorithm. Analyses included root mean square deviation (RMSD), root mean square fluctuation (RMSF) and structural snapshots.

### 4.11. Statistical Analysis

All results were expressed as the mean ± standard deviation (SD), based on triplicate measurements from three independent experiments. Differences between groups were assessed using one-way analysis of variance (ANOVA), followed by the Student–Newman–Keuls post hoc test. Data analysis was performed using GraphPad Prism 8.0 software, and results were considered statistically significant at *p* < 0.05.

## 5. Conclusions

The RQ18 peptide exhibits potent anticancer activity, particularly against murine and human melanoma cells, while showing lower cytotoxicity toward non-cancerous mammalian cells. Its high selectivity index and dose-dependent cytotoxicity support a preferential effect on malignant cells. RQ18 induced predominantly apoptotic cell death, as demonstrated by flow cytometry, and this response was accompanied by early intracellular oxidative alterations, changes in mitochondrial membrane potential, and activation of the executioner caspases-3/7. The temporal association among redox perturbation, mitochondrial dysfunction, and caspase activation suggests that oxidative and mitochondrial alterations may contribute to the apoptotic response induced by RQ18 in melanoma cells. In B16F10-Nex2 cells, the convergence of cytotoxic, oxidative, mitochondrial, and apoptotic responses provided a strong basis for further mechanistic investigation of RQ18 activity. Molecular dynamics simulations further supported these findings by revealing stable interactions between RQ18 and membrane models resembling those of cancer cells, together with the maintenance of its α-helical structure and strong electrostatic affinity for anionic lipid components. Collectively, these results indicate that the anticancer activity of RQ18 is closely associated with its structural and physicochemical properties, which may favor interaction with negatively charged melanoma cell membranes and trigger downstream oxidative, mitochondrial, and apoptotic events. These findings highlight RQ18 as a promising candidate for peptide-based melanoma therapy and provide a robust foundation for future in vivo pharmacological evaluation, including studies of antitumor efficacy, pharmacokinetics, biodistribution, and safety.

## 6. Patents

The patent application regarding the sequence and biological activities of RQ18 was made to the Instituto Nacional da Propriedade Industrial—INPI, Brazil, under the number BR1020190052031.

## Figures and Tables

**Figure 1:**
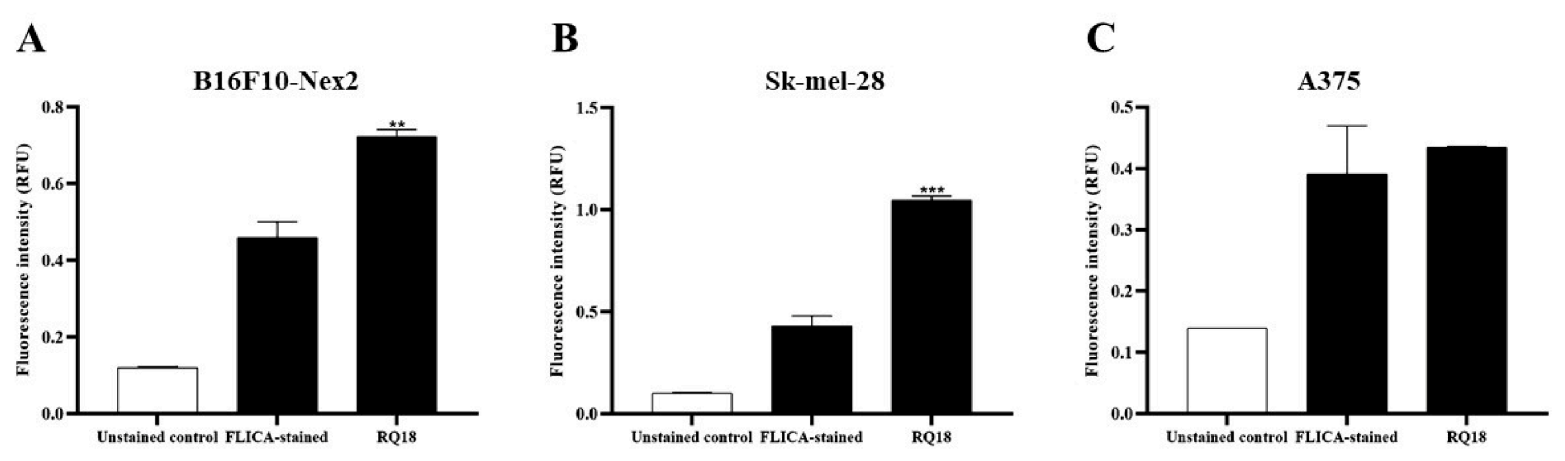
RQ18 induces caspase-3/7 activation in melanoma cell lines. Caspase-3/7 activity was assessed using CaspaTag^TM^ and the FLICA assay in B16F10-Nex2 (**A**), Sk-mel-28 (**B**) and A375 (**C**) melanoma cells following treatment with RQ18. Fluorescence was measured using a microplate reader and expressed as relative fluorescence units (RFU). Data are presented as the mean ± SD. Statistical significance: ** *p* = 0.01; *** *p* = 0.001; ANOVA.

**Figure 2:**
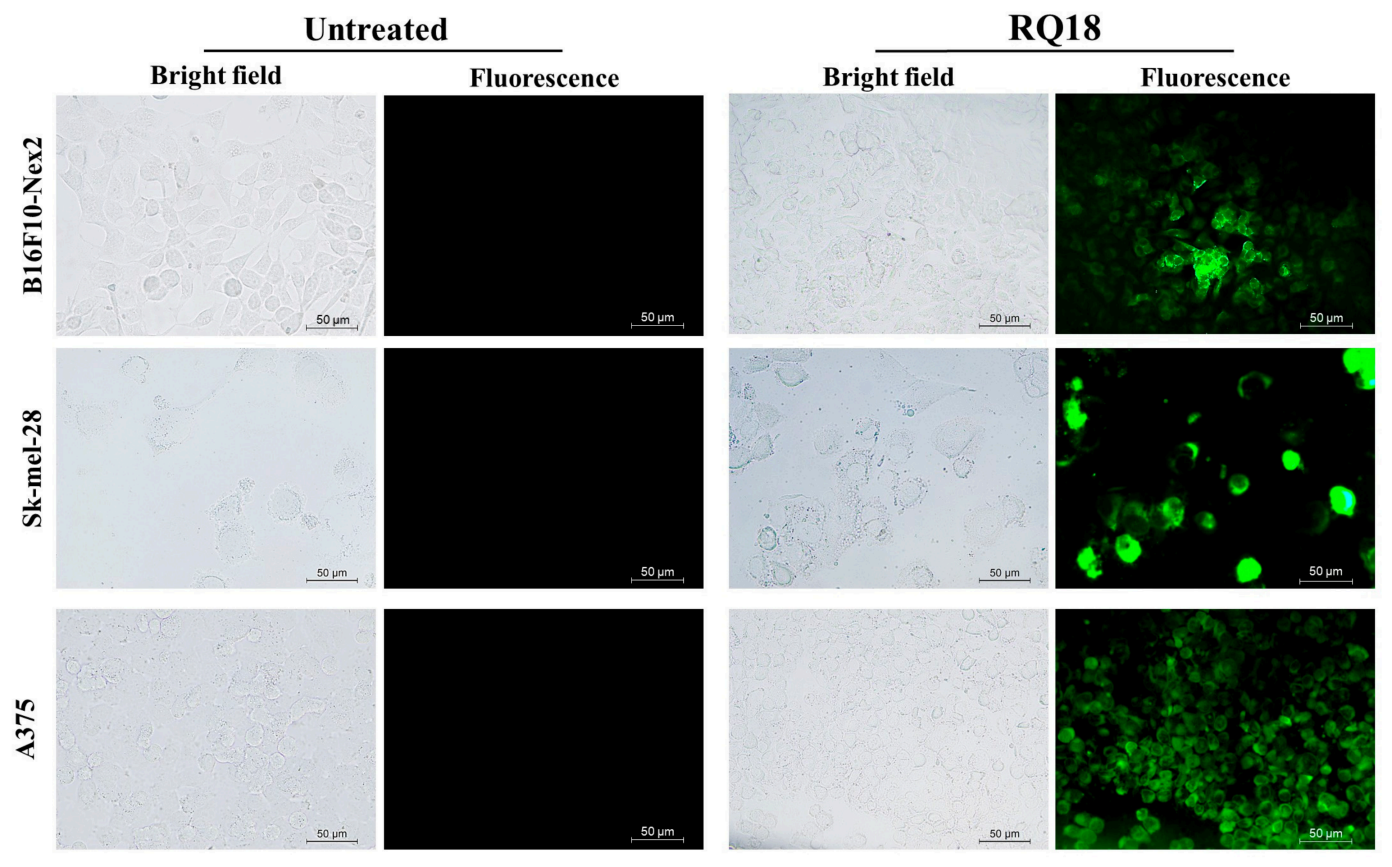
RQ18-induced intracellular oxidative activity in melanoma cells. Representative bright-field and fluorescence microscopy images of untreated and RQ18-treated B16F10-Nex2, Sk-mel-28, and A375 cells after 1 h of treatment at their respective IC_50_ concentrations. Cells were incubated with DCFH-DA (20 µM·mL^−1^) for 30 min and analyzed by fluorescence microscopy. Untreated cells showed no detectable green fluorescence, whereas RQ18-treated cells exhibited a marked increase in fluorescence intensity, indicating enhanced intracellular oxidative activity. Scale bar = 50 μm; 40× objective.

**Figure 3:**
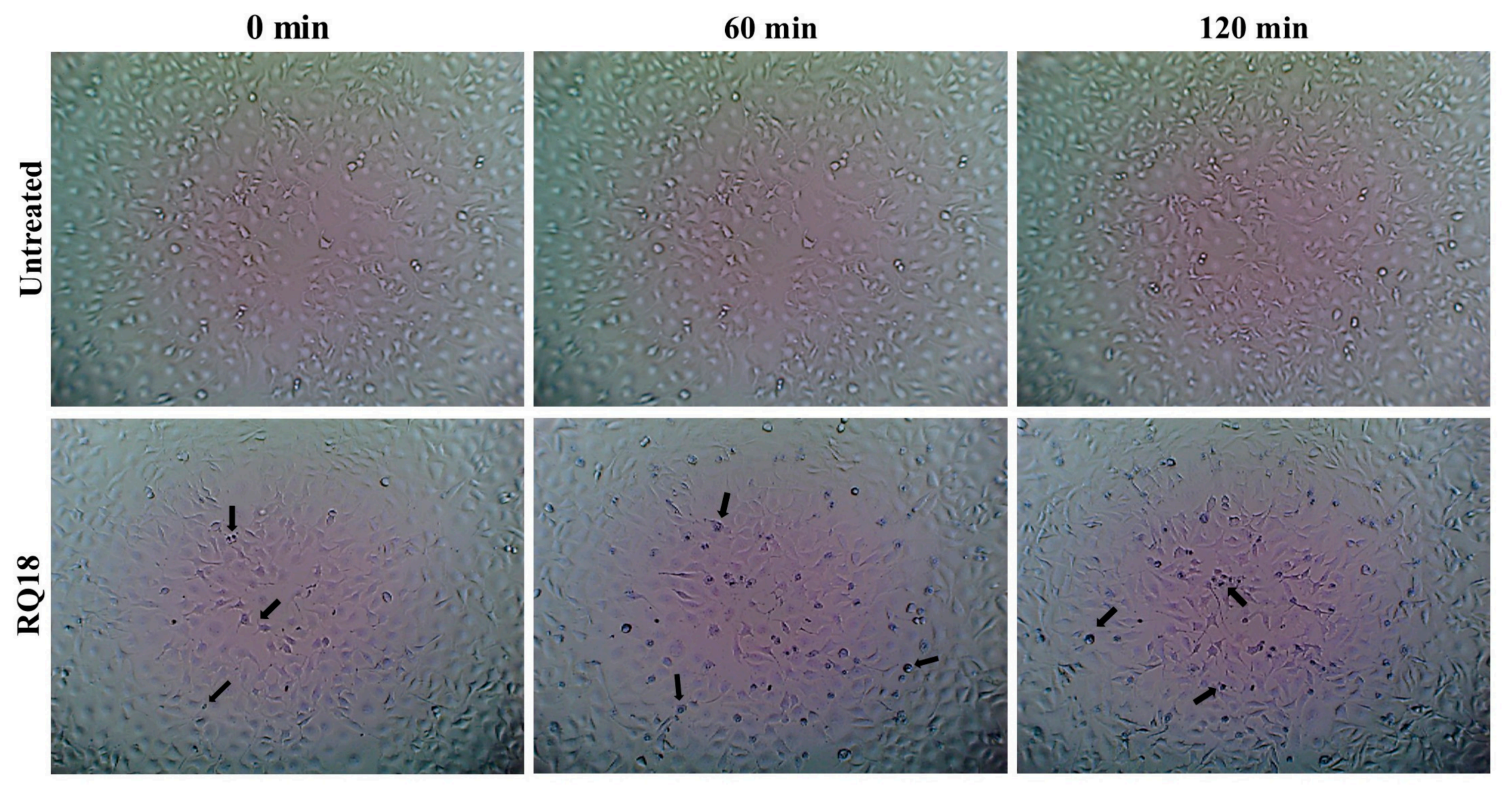
Morphology of untreated B16F10-Nex 2 cells and cells treated with RQ18 at the IC_50_ concentration of 4.4 µM·mL^−1^, observed at 0, 60, and 120 min. Arrows indicate some cells with chromatin condensation.

**Figure 4:**
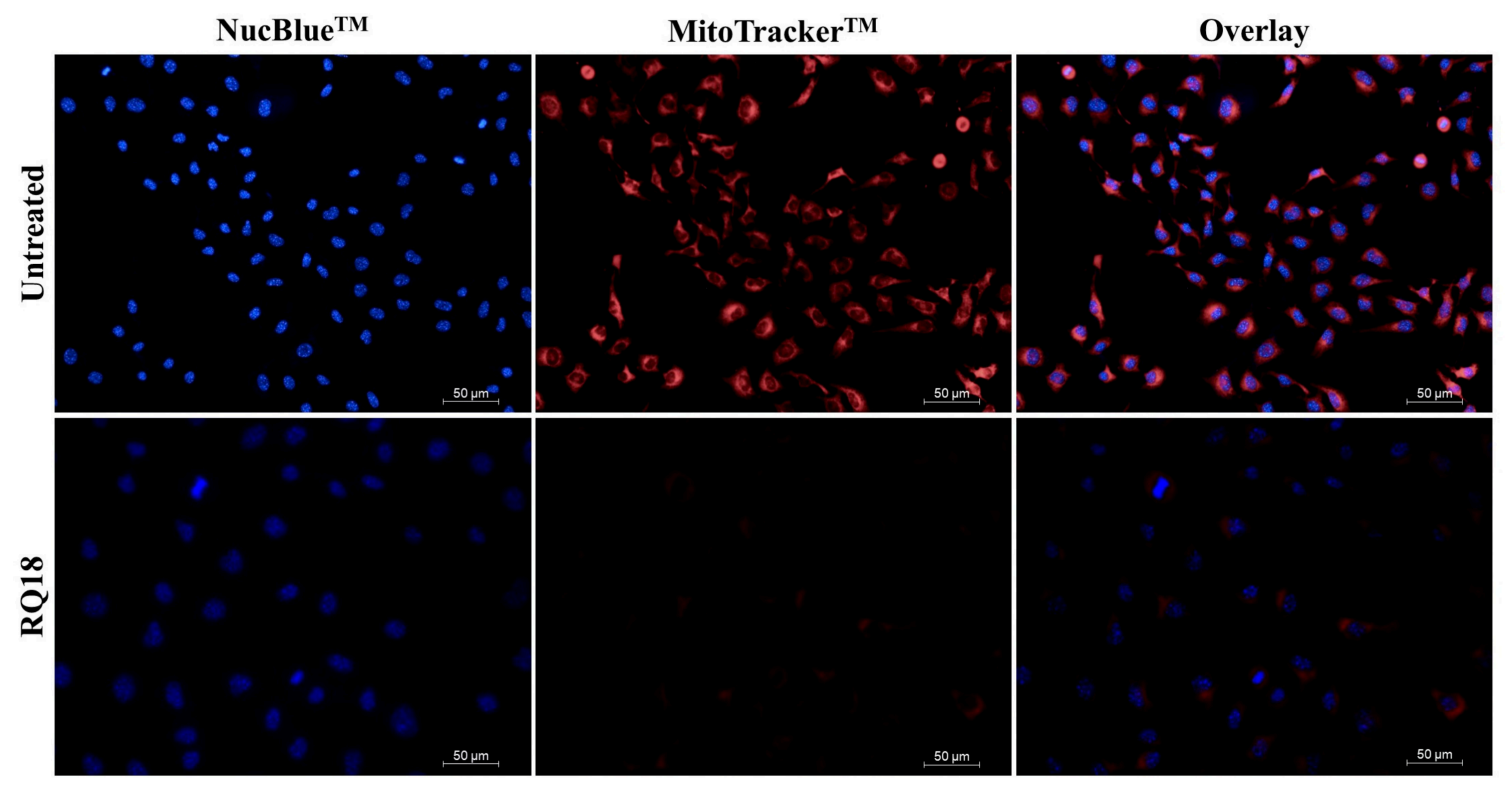
Morphological alterations in the mitochondria of B16F10-Nex2 cells treated with the RQ18 peptide at IC_50_ (4.4 µM·mL^−1^), compared to untreated control cells, after 24 h. Nuclei were stained with NucBlue™ (blue) and mitochondria with MitoTracker™ Deep Red (red). Scale bar = 50 μm, 40× objective.

**Figure 5:**
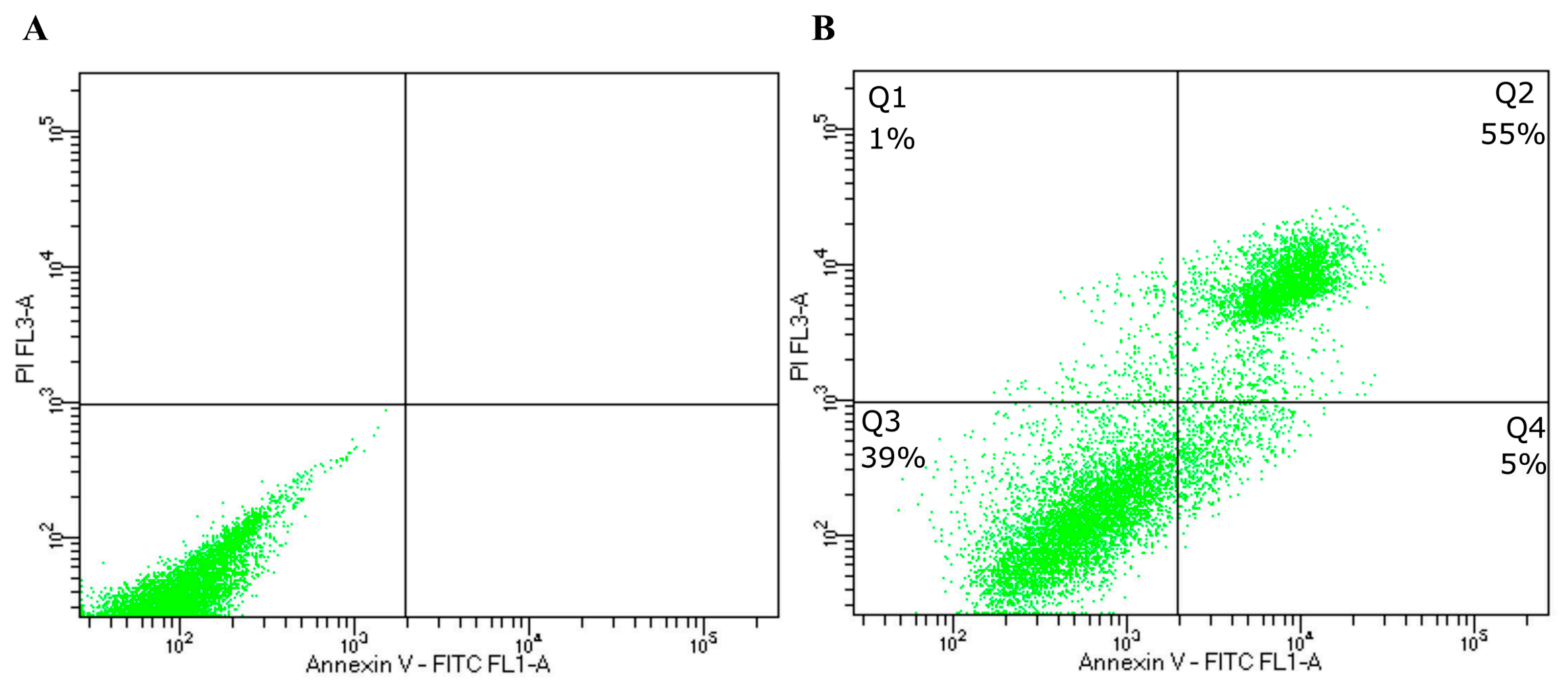
Representative dot plot (flow cytometry) of B16F10-Nex2 cells treated with RQ18 at IC_50_ (4.4 µM·mL^−1^). (**A**) B16F10-Nex 2 untreated control cells and (**B**) RQ18-treated cells. The axes represent fluorescence intensity for PE-PI (*Y*-axis) and FITC-Annexin V (*X*-axis). Quadrants Q1 to Q4 indicate different cell viability and apoptosis stages, with the majority of cells located in Q2 (late apoptosis) and Q4 (early apoptosis).

**Figure 6:**
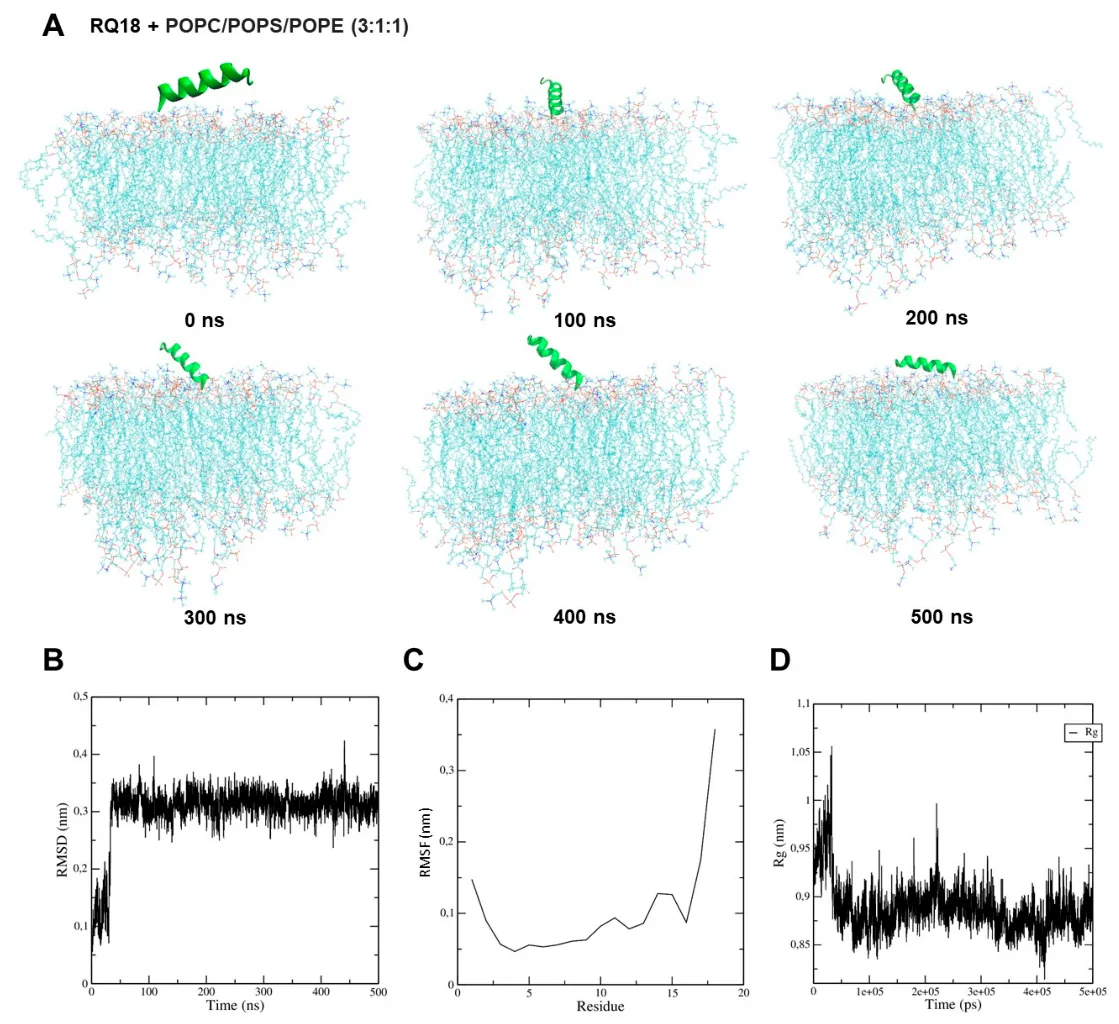
Molecular dynamics simulations of the RQ18 peptide in a POPC/POPS/POPE (3:1:1) lipid bilayer system (**A**). Simulations were performed using bilayers with an area of 75 Å^2^ over 500 ns, with snapshots taken at 100 ns intervals. The initial peptide structure was positioned approximately 0.35 nm from the membrane bilayers. The RMSD (**B**), RMSF (**C**) and (**D**) Rg of RQ18 were calculated for each simulated system.

**Table 1:** Prediction of the anticancer activity of the RQ18 peptide.

Predictors	Prediction Score
ENNAACT	100%
dbAMP	99.7%
ACPred	99.4%
MLACP2.0	90.2%
ACPP	Anticancer
AntiCP2.0	Anticancer

**Table 2:** Anticancer and cytotoxic activity of RQ18.

Cell Line	IC_50_^a^ (µM·mL^−1^)	SI ^b^
B16F10-Nex2 (Murine melanoma)	4.4	15.72
Sk-mel-28 (Human melanoma)	11.58	6
Hela (Cervical cancer)	15.58	4.45
MCF-7 (Human breast cancer)	120.6	0.57
A375 (Human melanoma)	59.99	1.15
FN1 (Human fibroblast)	23.08	-
RAW 264.7 (Murine macrophage)	69.2	-

^a^ IC_50_ was defined as the concentration of the peptide that inhibited 50% of cell viability, determined by the MTT assay. ^b^ SI (selectivity index) was calculated as the ratio of the IC_50_ value for RAW 264.7 to the IC_50_ value for each cancer cell line.

## Data Availability

The original contributions presented in this study are included in the article. Further inquiries can be directed to the corresponding author.

## Abbreviations

The following abbreviations are used in this manuscript:

ACP	Anticancer peptides
AMP	Antimicrobial peptides
FBS	Fetal bovine serum
RMSD	Fetal bovine serum
RMSF	Root mean square fluctuation
Rg	Radius of gyration
DMSO	dimethyl sulfoxide
MD	Molecular dynamics
